# A Wandering Supernumerary Tooth

**Published:** 1884-10

**Authors:** E. S. Talbot


### A WANDERING SUPERNUMERARY TOOTH.

BY E. S. TALBOT, M. D., D. D. S.

The following case, noticed by Prof. E. Fletcher Ingals, is of uncommon interest:

"Recently, in examining a patient who had for some time been troubled with nasal catarrh, I found on the floor of the left naris, four centimeters back from the nostril, a hard substance, feeling, when touched with the probe, like bone. On seizing this with forceps the patient experienced severe pain, like that caused by striking a sensitive tooth, and for several hours afterward he suffered from pain like a severe toothache.

"The pain caused by touching this body was so exquisite that it was necessary to produce complete anæsthesia before a thorough examination could be made.

"Dr. R. H. Lull administered ether for me, and I then engaged the foreign body in the snare which I use for nasal tumors, and drew it out, when it was found to be a supernumerary tooth, resembling closely the canine, and measuring two centimeters in length.

"About five millimeters of the tip of the root had been exposed in the nasal cavity, and below this the tooth was covered with soft tissue, which was adherent to it down to the crown. The dentine of the crown was perfect, excepting a small perforation at its apex, but within the tooth was decayed.

"How long this tooth had been projecting into the nasal cavity could not be determined, but it must have been a considerable time, for the portion of the apex of the root which was uncovered had lost by erosion about a millimeter in thickness from its entire circumference.

"It is comparatively rare for teeth to grow in the roof of the mouth or nasal cavities, yet several such cases have been met with. In nearly all instances the 'wild tooth,' as it is called by the laity, is found to be a supernumerary tooth, or a misplaced canine."

We were invited to examine the patient through Dr. Ingals. We found him an American, æt. 19, a book-keeper by profession. The superior maxillary was somewhat contracted, the anterior teeth overlapping and projecting at the median line. The roof of the mouth was high posteriorly, the alveolar process and mucous membrane thickened anteriorly. From the appearance of tartar and green stain upon the apex of the crown, it must have occupied a diagonal position in the hard palate, the crown being in the roof of the mouth, pointing toward the central incisors, and the apex of the root in the naris pointing posteriorly. A ring of tartar encircled the crown from about three millimeters below the upper surface to nine millimeters above the lower surface, and that part of the crown below the ring was covered with a fungous growth, which proved that that part only was exposed in the mouth. The tooth had decayed, because of the imperfectly formed apex of the crown, until the pulp had become exposed and death resulted, causing alveolar abscess, the sack of which came away with the tooth. The formation of the abscess causing pressure upon the hard palate, together with the burrowing of the pus through the palate, produced necrosis of the same. The pressure of the tongue upon the crown of the tooth forced the apex through into the naris.

